# Ovarian Tumors

**Published:** 1862-04

**Authors:** Wm. H. Byford

**Affiliations:** Professor of Obstetrics and Diseases of Women and Children, in the Medical Department, Lind University, Chicago, Illinois


### CHICAGO MEDICAL EXAMINER

N. S. DAVIS, M. D., Editor.---FRANK W. REILLY, M. D., Ass't Editor.

VOL. IV.] APRIL, 1862. [NO. IV

### Original Contributions.

ARTICLE IX.

### OVARIAN TUMORS.

By WM. H. BYFORD, A. M., M. D.,

PROFESSOR OF OBSTETRICS AND DISEASES OF WOMEN AND CHILDREN, IN THE MEDICAL DEPARTMENT, LIND UNIVERSITY, CHICAGO, ILLINOIS.

(Continued from March Number.)

The manner of applying the pressure is of the greatest importance; the apparatus should be permanent, and exert as much force as the patient can bear without too great pain, fever, derangement of the abdominal viscera, or other indications of too acute a degree of inflammation in the cyst or damage to some organ. It should be applied to the tumor as nearly as possible, and the forcible pressure should be exerted alone upon the collapsed mass, so as to crowd it back against the sacrum, lumbar muscles, spine and other hard parts of the posterior wall of the abdomen. In order to do this properly, after the fluid is evacuated as completely as possible, we should examine the abdomen minutely so as to ascertain, as clearly as possible, the position of the collapsed cyst. This will usually be a little more to one side than the other, and may, generally, easily define its shape, and get a good idea of its size. We should now construct a compact compress, corresponding in shape and size with the shape and size of the evacuated sac, the compress should be embraced by solid wood or tin outside. The compress can be made of hair, gum elastic material or napkins. If of the latter, they should be well stitched together, so that there can be no shifting in their position. After attaching the soft portion of the compress to the hard firmly, so that any pressure upon the latter may be exerted unvaryingly upon the former; it may be placed immediately over the tapped tumor, and pressure applied from a direction to press it against the hardest part, bearing on the posterior walls of the abdomen or pelvis. An attentive examination of the tumor under the pressure of the instrument will inform us pretty accurately as to the efficiency, completeness and direction of the pressure of the compress. The compress may be managed better by a belt of soft, but firm leather, to surround the body in such a place as to press over the centre of the compress. The power and direction of the pressure may be regulated thoroughly and at will, by subjecting it to a tourniquet screw pressure from the belt. Of course, there must be thigh and shoulder straps to the belt, in order to keep it from slipping up or down. When we have adapted these simple contrivances, we should turn the screw to such a degree as to press strongly as the patient can bear, and with it thereafter regulate the pressure as we may judge best. Having thoroughly satisfied ourselves of the appropriate adaptation of the aparatus, we should wrap the whole abdomen agreeably tight, from pubis to sternum, with a flannel roller. We should every day remove the flannel roller, and examine the com press and belt, to be sure that they are not disarranged, and if in the least so, we should re-adapt them. We may tighten the screw or loosen it each time, or allow it to remain untouched, as the case may be. The greatest care should be taken not to produce too great pressure with this compress. It should be loosened when chilliness, febrile excitement, or other general signs of distress are added to local pain; it may be tightened soon as the symptoms decline.

This mode of applying pressure, I think, is much more efficient and manageable than the plan recommended by Mr. J. B. Brown, the accomplished surgeon of female diseases and injuries, of London. His plan is to make a graduated compress of napkins, so as to fit the top of the pelvis, and after applying it over the tumor so as to press down into the pelvic cavity, and against its back part place over the whole a broad bandage tightly fastened, from pubes to sternum. With this appliance, we cannot always be accurate in the extent, position and rate of the pressure, and, consequently, much more skill and experience are necessary in its application. Its success hence is much more frequent in Mr. Brown's hands, than it has been with the profession generally. I am not aware that Mr. Brown teaches the necessity of pressure to all the collapsed tumor, but understand him to make most of his pressure at the origin of the tumor—the ovarian region. The tumor when collapsed by tapping, after great distention, seldom sinks anything more than partially into the pelvis; the long exercised traction upwards generally lifts the ovary of that side above the pelvis, and thus we may generally somewhat accurately fit our means to its slope and position. An objection Mr. Brown thinks sometimes applied to pressure, is the presence and great aggravation of prolapsus uteri. This objection it will be apparent, is very much more applicable to his mode of causing it, than the one I recommend. Multilocular tumors may be cured in this, perhaps more frequently, than any other way besides extirpation; for the pressure may be made to bear upon, and greatly influence the development of the small cysts that are not evacuated by pressure. I have more than once evacuated several sacs through one opening in the abdominal walls, by partially withdrawing the trochar, and directing the point toward a full sac, after the one first pierced had been evacuated. This should be attempted in a multilocular tumor before we use pressure; and it is allowable, I think, to introduce the trochar in several places where there are a number of cysts that cannot be reached by the instrument from one point. I would not be understood as advising a reckless use of the trochar in these many-cysted ovarian tumors; but after we have decided from the circumstances of a careful examination of a given case that tapping and pressure is the treatment, we risk nothing, I think, in being thorough in our efforts to evacuate as nearly as possible, all the sacs. The bad effects arising from tapping and pressure, are inflammation and its consequences. When there are symptoms of severe acute inflammation, the pressure should be removed, and leeches, cathartics, etc., should be employed to moderate or remove it. If the inflammation is in the sac, we should wait until all the acute symptoms subside, before the pad or compress is placed again; if, however, we can satisfy ourselves that the inflammation is in some other part distressed by the pressure, by varying the direction of the pressure, provided, we can include the tumor under it, we need not wait until all the acute symptoms have vanished. I have a better opinion of this kind of treatment when carefully managed and watched, than any other, except the complete extirpation of the ovary. Another plan of obliterating the sac of ovarian tumor is to first evacuate, and then inject it with some substance calculated to induce inflammation in it, which, by its adhesive or destructive processes, may completely effect this object. A large number of cases are reported cured by this plan of treatment. For obvious reasons, it is almost exclusively confined in its usefulness to the unilocular variety. Under certain circumstances only, can we expect to reach more than one cyst at a time with the trochar and injections. When a cyst is simple, the patient in good health, and we succeed in properly managing the operation, there is not a great deal of danger in it, and we may reasonably hope for benefit from it. The most simple, and I think, effective mode of operating, is to first draw off nearly all the fluid, except say, one or two pounds, as well as we can judge of it, with a large trochar. After this is accomplished, we should pass an elastic catheter, or other flexible tube, through the canula of the trochar to the bottom of the cavity. With a hard rubber syringe, we may inject the medicine, whatever that may be, through the catheter into the interior of the cyst. By using this elastic tube, there is no danger of failing to carry the material to the part we desire to reach, without its coming in contact with anything else, or being decomposed before it arrives at its destination. The formula for this kind of injections are numerous, and several different substances used. Iodine seems now to be the substance generally employed. Dr. SIMPSON recommends several ounces of the tincture. Six ounces is probably enough to use at one time. I have used on several occasions six ounces of a mixture containing one scruple of iodine, two scruples of iod. potass to the ounce of water. This is certainly iodine enough, if specific in its influence to cure any tumor. My plan is to allow it to remain in the sac, instead of removing any of it.

Iodism is likely to occur to a slight extent, but to be the source of no considerable inconvenience. If it should be thought best to remove a part, or the whole of the iodine, the better way to do it is to pump it out through the tube, by means of which it was introduced, instead of squeezing it back through the canula of the trochar. This plan of extracting it, precludes the possibility of allowing any contact with the peritoneum; which in the event of disarrangement of the canula, might otherwise take place. Although ordinarily, no great amount of acute inflammation takes place as the effect of this injection, yet we should remember that it sometimes does proceed to a dangerous extent, and be upon our guard with the means necessary to prevent a fatal degree. In fact, it would be negligence on our part not to watch with solicitude all the most trifling operations upon an ovarian cyst. It may be asked whether iodine is the best substance to use as an injection in such cases? Although I have to some extent fallen in with the fashion of using iodine, I cannot resist the conviction that there are substances that would do as well, against which, some objections that apply to iodine could not be urged. Iodine operates promptly upon the organism when introduced in this way, by being absorbed and taken into the circulation; yet I think, there can be but few who desire any thing more than its local effect upon the inner surface of the sac. Alcohol, wine, brandy, in fact any local stimulant whose general effect after absorption is more transient, as well as less powerful, would perhaps answer just as well. It cannot be that the internal effect of iodine upon the kidneys and other organs of excretion can enter largely into its good effects, for if such were the case, it would be better given by the stomach. Injection of iodine was regarded several years ago as the most eligible mode of treating this affection, because of its comparitive safety and frequent success; but there can be no doubt that it was overrated, and now the profession is less ready to trust it. I believe it to be both more dangerous and less efficient than pressure after tapping. This is not in accordance with the opinion of Dr. SIMPSON I believe. I have lately known of a case having been treated with iodine injections combined with pressure. I speak of this case to warn against similar proceeding, for it is plain, upon a little reflection, that if the pressure is properly applied, it will so lessen the cavity of the cyst as to endanger the effusion of the iodine, through the puncture in the sac, into the peritoneal cavity, and thus induce a fatal peritonitis. And if pressure is to be used, we should wait for two or three days after the injection.

The last and doubtless most effectual plan for obliterating the sac, is the establishment of a fistulous opening, communicating with the peritoneal cavity, or the external surface directly or indirectly, through the vagina or rectum. This plan is also the most dangerous plan, resulting in a large number fatally. Quite a difference in the effects, both remedial and morbid may be remarked in the different places for the fistulous opening. When properly and carefully managed, the opening in the peritoneal cavity is productive of least harm, and less likely to be followed by a cure. The opening in the vagina is more effective, and the direct opening through the abdominal walls both more efficacious and more hazardous, than any of the others. When a communication is perfected and perpetuated between the cavities of the tumor and the peritoneum, the surface of the latter being a better absorbing surface, the contents are absorbed, thrown into the circulation, and eliminated by excretion through the kidneys and alimentary canal. This process being carried on more rapidly than the secretion by the tumor, the latter is allowed to contract more and more, until its secreting surface is wholly lost, and indurated tissue is all that is left behind to mark its former existence. Some very important precautions are necessary to such happy results as will appear by an attentive consideration of the subject. It is found for instance, that sometimes the contents of the tumor are poison to the peritoneal lining of the abdomen, and therefore fatal inflammation may result from its effusion into the cavity. We cannot say without an inspection of the fluid, whether this is likely to occur upon performance of an operation or not, and I fear that we can by that means arrive at only a presumption upon the subject. In evacuating for the first time, these growths we find, occasionally, clear, transparent, good, innocent looking fluid, begin to flow, when as the flow continues, the latter part looks darker, grumous, and ill-conditioned; now, whether we might not be deceived upon inspection is a matter of question, and really furnish a virus to the surface of the peritoneum, instead of the bland albumen of the healthy ovarian tumors. However this may be, we do know from cases placed on record, by Dr. SIMPSON particularly, and observed, not unfrequently, that these tumors do sometimes burst into the abdominal cavity, and disappear, without any bad symptoms, so that we are justifiable in hoping the artificial opening may result well. Dr. SIMPSON recommends (and it is certainly the most sure way, although, as I have remarked, we must under all circumstances, be in doubt,) prior to opening communication with the peritoneal cavity, that we tap the tumor, and remove some of the fluid for examination, and if it is the ordinary bland, mucilagenous, transparent substance found generally after first tapping, he assures us we may proceed to the operation unhesitatingly, or rather may keep the puncture in the sac open afterwards, instead of allowing it to close up, as it usually does. This is done by, in the first place, not removing nearly all the fluid from the sac by tapping, but allowing enough to remain to keep it partially distended; and in the second place, every twenty-four hours so to press upon the tumor, as to well up the fluid through the opening in the sac, and thus break the slight adhesions which may have formed between the edges of the wound, and allow it to escape into the peritoneum. Dr. Simpson thinks this is the safer way, so far as the dangers from the operation is concerned, but as will be seen, not so certain of accomplishing the object. He has cured cases in this way. The most effectual and the most dangerous way is, to cut down upon the tumor, and remove a piece from its wall large enough to insure patency, withdraw a part of the fluid, and then close the wound in the abdomen, and allow the rest of the fluid to flow into the peritoneal cavity, thence to be absorbed. The immediate danger in this operation is, that of dividing some of the blood-vessels, which ramify through the walls of the tumor, and thus allow internal hemorrhage to take place. To avoid this, it is recommended by Mr. Brown to draw out, examine, and divide, only that portion which is clear of vascular ramifications. Others have recommended to tie any branch large enough to bleed. There is but little doubt that the precaution recommended by Mr. Brown would be sufficient to avoid that difficulty. The large wound through the peritoneum makes the chance of inflammation in that membrane greater than the mere puncture of the trochar. Upon the whole, I think I should prefer Dr. Simpson's plan of keeping the opening made by the trochar in the tumor patent, by frequent well directed manipulation. It ought to be practiced I think oftener than every twenty-four hours; as often as every twelve, for the first two days. It will probably be found upon extensive trial, that it may not be always practicable. Should there be adhesion at the point where the trochar passes, it would necessarily fail.

The plan of making a fistulous opening externally, is more practicable perhaps than the one just detailed, from the consideration that it is more manageable.

The operation is simple, and not attended with much immediate danger; the danger coming in the shape of acute inflammation soon after the operation, or exhausting suppurative inflammation, and its attendants. Mr. Brown, who has given it a more extensive trial than anybody else, selects a point midway between the umbilicus and the anterior superior spines of the ilium of the side in which tumor originated. His plan is to make an angular incision at this point down to the peritoneum, dissect up the angle from that membrane so as to completely expose it, evacuate the tumor through this exposed part with a trochar, stitch the sac to the sides of the opening, enlarge the puncture in the cyst, and keep it open by a pledget of lint or other substance as he finds most convenient. Others cut down to the peritoneum, at a point midway between the umbilicus and symphisis pubis, stitch the sac to the sides of the wound, and keep open by lint or stomach tube. Care should be taken, especially if the contents of the sac should have a suspicious appearance, to prevent it escaping into the peritoneal cavity. Often there is adhesion at this part when the stitches will not be necessary. This opening should be kept patent until the cavity of the cyst is lost by contraction, inflammatory adhesion, or granulation, or all these combined, which is probably the common mode of their disappearance. Some difficulty will be found in doing this; there is such a strong tendency in the wound to contract and heal up by granulation. If necessary, we may from time to time, somewhat enlarge it with the knife, and we should not allow it to close until the discharge has entirely ceased. From what I can see of the dangers of this operation, they are very little, if any, less than from ovariotomy; and I should not feel induced to resort to it, unless it were in a simple cyst, where tapping, injection of iodine, or the use of pressure had entirely failed, or where after exposing the cyst ovariotomy, was found impracticable, from extensive adhesions. This, I have done in one instance. The adhesions were so extensive, that the cyst could not be removed; in fact, they seemed to be about universal, the incision was small, only admitting two fingers, the sac had adhered at the point, where the opening was made, so the incision was all that was necessary in the way of an operation. The patient died of acute peritoneal inflammation in three days afterwards. A post mortem examination revealed extensive inflammation of the sac and peritoneum. I am inclined to try a modification of this operation if an opportunity offers in a patient who is not willing to submit to more severe operative procedure, and that is to perforate the tumor with a trochar half an inch or more in diameter, and through the canula, introduce a stomach tube, and leave it there, withdrawing the canula over it. The tube may be left until peritoneal inflammation glues the sac to the abdominal walls, if adhesion does not already exist. After this, the tube can be withdrawn, and re-introduced after cleaning, or, if it will not "go," one somewhat smaller used in its place.

Professors KIEVISH and SCANZONI, of Wurtzberg, are warm advocates of a fistulous opening through the vagina into the tumor, and keeping it open until the same obliteration takes place that was spoken of, as occurring in the case of opening through the front walls of the abdomen. SCANZONI operated on fourteen cases; eight resulted in a perfect cure; in two, the fluid collected again in a few weeks, one died of typhus fever two months after, and three were lost sight of. In none of the fourteen did death occur as a consequence of the proceeding. He mentions one case only, in his whole experience, in which death occurred from peritonitis, and that was Prof. KIEVISH's case. SCANZONI admits its danger, but shows quite a favorable opinion of it. Dr. WEST gives three cases of his own, two of which were cured, but had formidable inflammation; the third died, not as an effect of the operation, but from something else, which he does not state. SCANZONI taps with a trochar through the vagina, and allows the canula to remain until the cure is effected. This, of course, occupies a variable time; the tube was withdrawn by SCANZONI by the eighth or tenth day in some cases. He says that some of his cases recovered without any sign of inflammation, or other inconvenience. Dr. WEST operates by introducing the trochar, and withdrawing the fluid, passing a number twelve catheter through, and removing the canula over the catheter. The catheter is allowed to remain until the cure is complete. Simple cysts are the only kind that can be cured by the fistula method, and the recommendation to tap once, to be sure that there is but one cyst, is a good recommendation. The cyst cannot always be reached from the vagina, and only in such cases as it is crowded down into the pelvis, so as to give obvious fluctuation in that canal, should we think of this operation.

The third object in the treatment, partial or complete removal of the diseased mass remains to be considered. Could the tumor always be removed when the operation is once commenced, one of the greatest objections to ovariotomy would be removed, and the question of the propriety of attempting it, would be very much simplified; but unfortunately the operation cannot only, not be finished in many instances when attempted, but it is utterly impossible in our present state of knowledge, to predict with the most favorable opportunities whether difficulty will arise or not. The only obstacle worth considering, if not the only one, to completion of the operation of ovariotomy, is the adhesion of the tumor to the abdominal walls or viscera so strongly and largely, that it is impossible to separate them, and if separated, to add very much to the dangers of inflammation. This obstacle and its dangers are so great, as to entirely deter many courageous surgeons from performing ovariotomy, or allowing it to be considered a legitimate operation; but notwithstanding the above facts when confined to appropriate cases, with appropriate tentative measures, care, and the avoidance as far as possible, of the objectionable features of the operation itself, in the modes of performance more particularly, and the desistence from or change in the operative procedure when much adhesion exists, I should unhesitatingly advise a resort to it.

I cannot help believing, or refrain from expressing the opinion, that very few operations of ovariotomy are performed in which there is not culpable neglect of the avoidance of some of the serious causes of disastrous consequences.

I shall be understood better when I bring together in one view some of the effects which generally intervene between the surgical violence of the operation, and fatal termination. They are 1st, peritoneal inflammation, 2d, hemorrhage from the peduncle, 3d, pyæmia, 4th, abdominal shock, 5th, exhaustion.

The danger from the above conditions is somewhat, in comparison with the order in which they are named, the most danger is to be expected from peritoneal inflammation, next from hemorrhage, etc. Now an operation ought never to be performed when possible to do otherwise in such a manner as to *increase* the risks of any one of the above conditions. Everything should be avoided, so far as at all practicable, that irritates the peritoneum in any shape, perfect security from hemorrhage should be an indispensable consideration. It is not supposable that with every possible improvement, the operation will ever become safe, but there is every rational position in favor of the assumption, that many deaths may be avoided by thus rigidly adhering to a determination to leave nothing undone to lessen all the above mentioned causes of death. Success in a great undertaking, notwithstanding bad management, should not encourage us to hope for a like purely fortunate result. The more good sense and science we can combine in an operation, the more likely we will succeed. Many good men do things they know hazardous, *when they could as well avoid them*, because they have been successful in the same way before. Dr. TYLER SMITH read four cases of ovariotomy before the London Obstetrical Society, which are published in the *Lancet* for September, 1861, in one of which he says, "the ligatures and stump were cut off as closely as possible, and the whole returned into the pelvis, with the expectation that they would be quickly enveloped in coagulable lymph, *so as to prevent injury*. The patient recovered without a single bad symptom, taking no medicine but thirty drops of laudanum. This woman recovered well, and will justify similar procedure when unavoidable, but the treatment was bad and cannot be justified; because Dr. SMITH could, and ought to have avoided the risk, which rendered the development of coagulable lymph to prevent danger from the unnecessary presence of his irritating ligatures. Mr. SPENCER WELLS very justly reprimanded him for it in a gentlemanly way, by saying that the procedure ought not to be imitated without more discussion. Before undertaking to avoid or prevent any of these untoward circumstances which result from ovariotomy, it will be necessary to examine more at length the immediate causes of them. And first of peritoneal inflammation. One of these causes, to a great extent, unavoidable, is exposure of the peritoneal cavity to the contact of air, and, in many instances, unnecessary handling or contact with the fingers, sponges, cloths, etc. The greater in amount, the longer duration either of these sources of irritation are applied, the more danger, notwithstanding immunity observed in cases where both were extreme. If contact of the atmosphere is slight, and its duration short, we must have less danger to apprehend than if the whole abdominal cavity were exposed for a long time, and it cannot be a philosophical conclusion, that a small wound two inches long, exposing but a small extent of the peritoneum, open but ten minutes, is as dangerous as an incision from sternum to pubis open for an hour. Much less is it rational to handle all the viscera, wipe them, and return them into the abdomen, and perhaps sponge out the whole cavity. That women have recovered from such rash acts, is no reason why they are proper, or allowable, when it is possible to avoid them. While this kind of causes of peritoneal inflammation cannot be wholly avoided, all that is possible, should be. The incision should be no larger than necessary, to permit the extraction of the collapsed tumor, and the hand should never touch the peritoneum when adhesions do not make an enlargement of the incision necessary, and the introduction of the fingers indispensable, then the enlargement of the opening should be carried to the least possible degree, and the handling as slight as necessary to accomplish the object, either to separate the adhesions or satisfy us that this is impracticable. Perhaps the return of the ligature into the abdomen is fraught with more danger than any other of the ordinary parts of the operation. Its contact with the peritoneum will irritate it, and awaken inflammation, unless in very fortunate cases, it is surrounded with fibrine, and thus the danger averted. It should be remembered, however, always, that it is a *danger averted*, and that the limit of the inflammation is the aversion of the danger, and that the presence of the ligature is the danger. But suppose the ligature not left in the peritoneal cavity, and consequently needs not to be surrounded by fibrine, but that it is left outside the abdomen at the wound, still its presence is a source of danger, from the strangulation it causes. Inflammation must take place to slough the ligature off, and is very likely to spread along the membrane into the abdomen, and become general when once it has a starting point. This last danger from peritoneal inflammation, from the use of the ligature, is as imminent when the clamp is the agent of compression or strangulation; and I think, when it is possible to do without either, we should do so. The inclusion of the ligature, although it may not light up extensive and rapidly fatal peritonitis, as a focus of phlegmonous inflammation it may generate pus enough to poison the blood, and induce pyæmic fever and exhaustion. It is most likely that pyæmia results more frequently in this, than any other way. But the inclusion of the ligature is the cause of the next most frequent condition effecting death, after ovariotomy, that is hemorrhage. However secure the ligature may be applied, apparently, there is no certainty that bleeding will not occur from the peduncle.

Dr. West introduces a table, which perhaps shows the subject in its proper proportion. In fifty-nine cases,

| | | | | |
|---|---|---|---|---|
| 29 | proved | fatal | from | peritonitis. |
| 13 | " | " | " | hemorrhage. |
| 8 | " | " | " | exhaustion. |
| 2 | " | " | " | shock. |
| 3 | " | " | " | abscess. |
| 2 | " | " | " | ulceration of intestines. |
| 1 | " | " | " | tetanus. |
| 1 | " | " | " | phlebitis. |
| 59 | | | | |

It will be seen that a large proportion of the deaths occur as the consequence of exhausting hemorrhage in the peritoneal sac, the patient bleeds to death internally. But if the hemorrhage should not be great enough to exhaust and cause death, as a hemorrhage, as an irritating substance, it may produce peritoneal inflammation, and in that way cause death. Ulceration of the intestines, in consequence of the ligature lying in contact with them, is another evil, resulting from the presence of the ligature in the abdominal cavity. The principle objections to the ligature, and which should warn us against its use, either internally or externally are, that it causes peritoneal inflammation by lying in contact with it in the cavity; by inducing strangulation when either internal or external, and sending forth the inflammation along this very susceptible membrane, thus causing it to become general; by inducing phlegmonous inflammation, exhausting abscess, and perhaps, also causing pyæmia, and lastly, the great uncertainty in securing the vessels of the stump against hemorrhage. I regard the dangers here mentioned against the ligature as adding so much hazard to the operation, that I could not think of making use of it in any shape, unless it seemed entirely indispensable in the particular case. If I should find this to be the case, I should be inclined to imitate the example of Dr. TYLER SMITH, by cutting it off close to the stump, and returning the whole, instead of leaving the end of it out of the wound, both to add to the extent of membrane touched by it, and consequently the amount of irritation; and to serve as a medium to admit a constant supply of air in the peritoneal cavity, and thus very materially promote suppuration and putrifactive decomposition of any retained effusions. More can be done fortunately, to prevent peritoneal inflammation and internal hemorrhage than any other conditions which are found to prove fatal by their presence. The shock which succeeds the operation, now that chloroform can be made to shield the nervous system against the effects of such extensive violence, is not so much to be feared, but should always be carefully guarded against. If inflammation and hemorrhage can be prevented by improvements in the operation, even in fifty per cent. of the cases, fatality will be very materially decreased. Whatever may be the teaching of experience as to the tolerance of, and recuperation from violent and rash procedure in the performance of so dangerous an operation, it cannot justify us, in making use of means or methods that common sense, physiology, and pathology, all combine in declaring more hazardous than other means and methods, which we can employ. Let therefore no more incision and exposure, or handling of the viscera be allowed than is absolutely necessary to extract the collapsed tumor; and discard scrupulously the ligature and clamp. After so much of an introduction by way of expressing my conviction that too much preventive measures cannot be employed, and that the operation is too often more hazardous than it need be, by following the examples of men who have succeeded in spite of rashness and carelessness, I am ready to describe what I consider the best method of operating. The best time for operating, is about the middle of the menstrual month of our patient, and if consistent with the circumstances of the case between the first of October and the first of June in this climate, but of course, all will depend upon the urgency of the symptoms in the case, as to this last item of time. The patient should not be too much prepared, as it is easy to get up functional disturbance, and thus with medicines intended to place the patient in more favorable condition for operation, get the system in a state the least calculated to resist the attack of traumatic disease. Above all, we ought not to disturb the abdominal organs with a cathartic, and if the stomach, liver, or other organs require a purgative, it should be given three or four days before the time to operate, and we should give them time to recover their tranquil tenor of function, before we venture upon it. Should the bowels be constipated, we may give an enema six or eight hours before hand, but not oil, or other cathartics. It is good practice to give a pretty full dose of opium an hour before we put the patient upon the table. This is all the preparation she needs, provided she is in good condition ot general health. Of this last, we should be sure, when practicable. We should at least satisfy ourselves that there is no predisposition to the suppurative, erysipelatous, or inflammatory diathesis. For three hours, she should be in a state of perfect physical and mental tranquility, if possible to secure it, and at the time, placed in a perfect anæsthetic condition before being removed from her bed, and without seeing any of our preparations. *We* should be prepared, by carefully passing in review the successive steps of the operation minutely and deliberately, performing the operation mentally first, making provision for all possible complications or embarrassments, and carefully placing everything we shall need, as suggested by this kind of reflection, just where it can be most handily available. We should next state to our assistants what we expect to do, in each successive step in the operation, and the part we desire them severally to perform, under all given circumstances and times of the operation. This complete understanding between all the parties who partake with us in the operation, will preclude the danger of confusion and possible imperfection in the procedure. We want a good scalpel, blunt-pointed bistury, large trochar, two large ligatures in needles, an ecrasseur, half a dozen silver pins three inches long, with moveable steel points to them, several large waxed ligatures, three or four small needles, armed with silver wire, a tenaculum, silk for artery ligatures, and well to have small artery forceps. This will constitute a pretty full operative armament. We shall also want two or three fine sponges of different sizes, a flannel roller large enough to make a binder for the patient, one pan of cold and warm water each, some adhesive plaster, and old cloths of linen for compresses. Our table should be firm, about two feet wide, six feet long, and conveniently high, covered with sufficient soft quilts not to be too hard, and placed near the lightest part of a large and airy room, brought to the temperature of about 70 Fahrenheit. When all the above preparations are made, we are ready to operate. We should have at least three intelligent physicians as assistants. One to act as main assistant, one to use the chloroform, and one as a general handy man.

Supposing our diagnosis to be as complete in all respects as possible, especially with reference to the nature of the contents of the tumor and the adhesions, etc., we may cause the bladder to be evacuated, etherize and place our patient on the table, on her back, with the limbs hanging over one end to the knee. We should stand at the end of the table in front of the patient, when the large protuberant abdomen will rise before us, and the part below the umbilicus completely exposed. With the scalpel, the operator should make an incision in the linea alba, midway between the umbilicus and symphisis pubis, about two inches long, through the walls of the abdomen, down to the tumor, but he should avoid opening the cyst with the knife. It will require some care to avoid the cyst, particularly as the aponeurosis is thin at this place; the strokes should be light, and an examination for the peritoneum after each touch of the knife, soon as near the proper depth has been reached. When the sac is exposed, we should plunge the trochar into it and evacuate it, and so far as possible, extract it through the wound. If the tumor is multilocular, this procedure will soon bring another cyst to the incision, when it should be punctured and evacuated, and thrown out through the wound, the next punctured, etc., until the whole is reduced sufficiently in size to be drawn entirely out. Should there be a solid portion at the base of the tumor so large as not to come out of the wound easily, we may enlarge the latter sufficiently. All this being done, the tumor lying outside the abdomen; the pedicle passing through the opening should be pierced on each side with one of the needles, with the large ligature, as near as possible to the tumor. This is for the purpose of giving us perfect command of the stump, after the tumor is separated. The chain of the ecrasseur should now be thrown around the peduncle, at the base of the tumor, leaving the stump long as possible, and slowly crushed through it. We should next place the stump in the wound, leaving its edge a little above the level of the skin, and by means of the silver pins one inch apart, surround them with the thread, and secure perfect adaptation of the lips of the wound. The pins should be made to enter the skin about an inch from the edge of the incision on one side, dip down as near to the peritoneum without touching it as we can well effect, and piercing the opposite lip, come out about an inch beyond the other side of the wound. If the edges gape at all, some superficial stitches with the fine silver wire should close this more completely. A compress of wet linen should be placed over the wound, and all surrounded by the flannel roller, the patient removed and placed in bed, and allowed to come from under the effect of the chloroform. This operation is simple, easily performed, and I think inflicts as little dangerous violence, if not less than any other operation, the details of which are given. The extent of the wound is the least possible for the purposes of it; no handling or even rough contact of the viscera is required; the peritoneal cavity is exposed in the smallest extent, and for the shortest time; and all the objections and dangers urged against the ligature around the stump or in the abdominal cavity, are obviated and avoided. The separation of the peduncle with the ecrasseur, and the placing the edge outside the wound, so that if hemorrhage does occur, it is outside, and is within the control, as well as observation of the medical attendent, lessen, it seems to me, very materially the *causes* of inflammation at least; and as a logical, if not actual sequence, the dangers of the operation.

Very many cases, however, which promise to be favorable for the above procedure, so far as as we can judge from examination before hand, as we proceed in the operation we will find cannot be terminated without much more difficulty, and in some, our procedure must be very materially varied from the above detailed plan.

The main, and almost only obstacle to the performance of this operation is adhesion of the tumor to the abdominal walls or viscera: the amount and firmness of adhesion will determine the extent and nature of the varying steps. The incision through the abdominal wall should be the same in all cases, and unless adhesion be at the point where the incision is made,

I would evacuate the presenting cyst, and remove it through the opening as far as practicable, and if the adhesions were small and easily overcome, do so by traction; or if to the intestines or omentum they might be separated, after bringing them under the eye through the wound, and we should thus proceed to evacuate separate cysts as far as practicable. Should this plan prove ineffectual, after persevering trial, the opening should be enlarged to four or five inches in extent, and then a careful and thorough inspection of the condition of adhesions be made, and if they are extensive and numerous, the idea of complete extirpation should be abandoned; but so much of the cyst or cysts should be evacuated and withdrawn through the wound as possible, without great violence. If it should be practicable to withdraw a considerable portion, or even if not, we should lay open that part at, or outside, the incision, and turn the sides over each edge of the wound, and through the incision in the cysts try, and if possible, evacuate the fluid from all the remaining cysts, and in this way as perfectly collapse the tumor as possible. If practicable, we ought not only to puncture the cysts that we cannot remove in this way, but remove a part of the partition between those remaining, so as to convert them all into one cavity. We should now separate all the tumor outside with the ecrasseur, first securing it, so that the portions remaining do not pass beyond our reach into the abdominal cavity. Bringing the edges of the amputated sac above the surface, it should be then secured by the pins as in the first proceeding, and the wound should be closed up, except to the extent of half an inch at the lower angle, which should be kept open by lint, or the insertion of a flexible tube. I should not feel inclined to use a great deal of violence in the separation of the adhesions, nor to ligate large surfaces, and leaving the ligatures in contact with the peritoneum. If none of the sacs can be removed, after puncturing and destroying, as far as possible, the partitions, we should keep the wound open as above directed. The after treatment should have reference to the danger from the shock, from which the system may not recover itself; peritoneal inflammation is by far the most dangerous; and where a part, or the whole of the tumor is left, inflammation of the cysts or hemorrhage from the amputated stump.

Opium liberally used, is the best means of guarding against the shock, and occurrence of inflammation. The patient should be kept fully under its influence for the first forty-eight hours, and then it should be slowly and gradually decreased in doses, so that its influence may be prolonged for two days more. Generally when the first four days are fully passed without symptoms of acute inflammation, the danger from that cause will have pretty nearly passed, and we should let the patient slowly come from under the influence of the opium. I need not argue or bring examples in proof of the efficacy of opium in preventing inflammation after operations; most surgeons rely upon it with a good deal of assurance. Some difference as to the quantity in different persons, to produce the same effect, will make it necessary to watch the effect of the first dose, before we can determine how much may be given at stated intervals. For the purpose of doing this, as well as obviating any other difficulty or danger that may present itself, we should remain with her, or leave some competent physician by her bedside for the first two days. Two-grain doses may be given every four hours—and I think solid opium best—and if this does not make a decided narcotic impression, the quantity should be increased. Mr. BROWN recommends small pieces of ice in the mouth to suck, as one of the means preventive of inflammation, and as they are very grateful, particularly the second day, I would join him in it. The bladder must be watched, and evacuated at least every eight hours. If the patient can do this spontaneously, it is well, if not, the catheter ought to be resorted to. Every two or three hours the dressing in the region of the wound should be inspected, lest a slow, but exhausting hemorrhage occur, and the chances of our patient be lessened. Although the ecrasseur generally suffices to prevent hemorrhage from the stump, it is not always so, as I have verified in one instance, at least. Should nothing threatening take place at the expiration of the third day, opium may be withdrawn slowly, by gradually lessening the dose, or lengthening the intervals for its administration, and from this time forward allow the patient to return to her ordinary regimen gradually. The diet for the first few days should be scanty. Unless some threatening symptoms appear, we need not continue rigidity in respect to diet longer than a very few days. Perfect quietude should be observed for a week at least, and then return to exercise should be slow, and by very gradual steps.

There cannot be any very definite directions given as to the treatment of inflammation, or any other of the effects of the operations, other than may be found with reference to them in any well digested treatise.

We must be watchful to perceive the very beginning of them, and energetic in the treatment. An incipient inflammation may be subdued much easier than a well established one. In instituting measures to combat inflammation or any of these effects, we must carefully consider the condition of our patient; there being no question that tonics and even stimulants with anodynes, are better adapted to cure inflammation than depletion in patients of this kind, under certain circumstances. It is only in the young and robust, well nourished at the time, that depletion can be extensively used; in moderation when early used, there are but few who will not be benefited by it, even when we must follow it with quinine and stimulants. There are a few chronic cases in broken down constitutions, emaciated, highly debilitated, and particularly dyspeptic with depraved secretions generally, that we must sustain from the time of the completion of the operation, in order to prevent inflammation and exhaustion, one or both.

---

Iron in Chlorosis.—M. Trosseau is now strongly of opinion that in those cases of chlorosis in which there is a tendency to tubercular disease of the lungs, preparations of iron, administered for some length of time, favor and hasten the development of the tubercles. It is, therefore, of every importance in treatment to distinguish between true chlorosis, and what he calls pseudo-chlorosis.—*Brit. Med. Jour.*

